# Recent innovations in fluorescence lifetime imaging microscopy for biology and medicine

**DOI:** 10.1117/1.JBO.26.7.070603

**Published:** 2021-07-10

**Authors:** Rupsa Datta, Amani Gillette, Matthew Stefely, Melissa C. Skala

**Affiliations:** a Morgridge Institute for Research, Madison, Wisconsin, United States; b University of Wisconsin, Department of Biomedical Engineering, Madison, Wisconsin, United States

**Keywords:** fluorescence lifetime, microscopy, fluorescence lifetime imaging microscopy, image analysis, perspectives

## Abstract

**Significance**: Fluorescence lifetime imaging microscopy (FLIM) measures the decay rate of fluorophores, thus providing insights into molecular interactions. FLIM is a powerful molecular imaging technique that is widely used in biology and medicine.

**Aim**: This perspective highlights some of the major advances in FLIM instrumentation, analysis, and biological and clinical applications that we have found impactful over the last year.

**Approach**: Innovations in FLIM instrumentation resulted in faster acquisition speeds, rapid imaging over large fields of view, and integration with complementary modalities such as single-molecule microscopy or light-sheet microscopy. There were significant developments in FLIM analysis with machine learning approaches to enhance processing speeds, fit-free techniques to analyze images without *a priori* knowledge, and open-source analysis resources. The advantages and limitations of these recent instrumentation and analysis techniques are summarized. Finally, applications of FLIM in the last year include label-free imaging in biology, ophthalmology, and intraoperative imaging, FLIM of new fluorescent probes, and lifetime-based Förster resonance energy transfer measurements.

**Conclusions**: A large number of high-quality publications over the last year signifies the growing interest in FLIM and ensures continued technological improvements and expanding applications in biomedical research.

## 1 Introduction

The fluorescence lifetime is the average time that a fluorophore remains in an excited state before returning to the ground state and emitting a photon. The fluorescence lifetime is sensitive to the environment of the fluorophore, including pH, temperature, viscosity, and binding state. Fluorescence lifetime imaging microscopy (FLIM) provides information on molecular interactions that are advantageous for biological and medical imaging. FLIM captures the decay time of fluorophores at each pixel of the image, and therefore, provides unique information compared to intensity (photon flux) or spectral (photon wavelength or energy) imaging of fluorescence. Unlike intensity-based measurements, lifetime is self-referenced and independent of the absolute number of photons. Therefore, FLIM is not corrupted by variations in intensity between pixels. Our previous review covered the fundamentals of FLIM instrumentation and analysis, along with applications to live cell and tissue imaging.^1^ The goal of this perspective paper is to provide updates of innovations in FLIM which we, as experts in the field, found impactful within the last year. These new developments have been categorized into advances in instrumentation, new approaches to FLIM data analysis, and recent applications of FLIM in biology and medicine. The developments covered in this perspective are not exhaustive and innovations in FLIM instrumentation, analysis, and applications are ongoing. For a more comprehensive review on FLIM, readers are encouraged to refer to other publications.^1^^–^^5^

## 2 Advances in Instrumentation

### 2.1 Fast FLIM Techniques

**Fig. 1:**
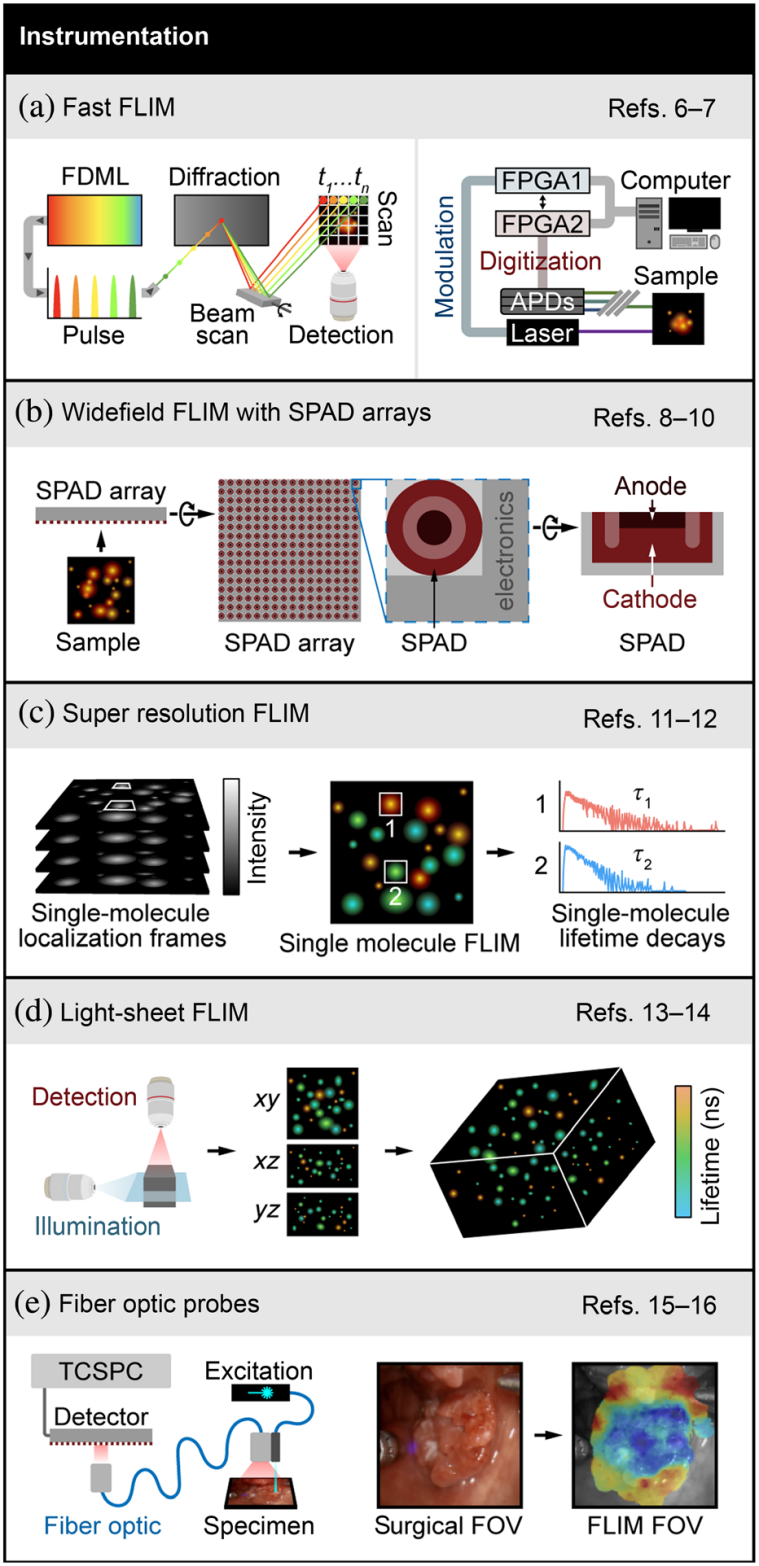
Advances in FLIM instrumentation. (a) Left panel: simplified schematics of SLIDE microscope that uses swept-source FDML laser that is pulse modulated such that each pulse is encoded both spectrally and in time, resulting in sequential pixel-wise excitation. This technique, along with a fast digitizer, achieves FLIM acquisition at high speed. Adapted with permission from Ref. 6. Right panel: simplified illustration of frequency domain lifetime imaging system implemented with FPGAs that modulates the excitation laser and digitizes the emissions using fixed gain APDs. Stylized image representing original publication with the permission of AIP Publishing.^7^ (b) Schematics of time-gated SPAD array consisting of SPAD photodiode and electronics to measure individual photon arrival times. Cross-sectional view of a SPAD showing key components—diode anode and cathode. FLIM systems implemented with SPAD arrays provide fast FLIM acquisition from large samples. (c) Super-resolved FLIM is acquired with SMLM techniques. Stylized illustration adapted with permission from Ref. 11 shows intensity frames including single-molecule localizations, single-molecule lifetime map, and histogram of photon arrival times for two indicated single-molecule localizations. (d) Simplified schematic showing FLIM with light-sheet illumination scheme that results in volumetric FLIM, adapted with permission from Ref. 13. (e) Simplified schematics showing fiber-optic-based FLIM system with TCSPC electronics for time domain FLIM data acquisition. Also shown are white light image of surgical FOV and FLIM FOV with augmented lifetime map overlay. Adapted with permission from Refs. 15 and 16. References to all relevant papers for each section have been noted.

FLIM instrumentation has seen major advancements in recent years. FLIM is typically characterized by slow acquisition speed because high photon counts are needed for accurate decay rate estimation. Thus recent efforts have focused on improving FLIM instrumentation to increase acquisition speed. Karpf et al.^6^ developed the spectrotemporal laser imaging by diffracted excitation (SLIDE) microscope that achieves high-speed imaging by employing an electro-optic modulator, and high-speed swept source Fourier-domain mode-locked (FDML) laser, the output of which is then passed through a diffraction element. This results in spectrotemporal encoding of each pulse such that the wavelength provides point scanning, and the temporal information is used for signal to pixel mapping. The fluorescence is then demodulated using a fast digitizer to generate FLIM images. Figure 1(a) (left panel) shows a simplified schematic of the SLIDE microscope. The authors report FLIM acquisition speed of a million pixels per second. However, the limitation of this microscope is the requirement of bright samples for generating high photon count rates and higher two-photon excitation powers (∼10 to 100 mW). Alternatively, field programmable gate arrays (FPGA) can provide a fast FLIM modality in conventional FLIM collection schemes [Fig. 1(a), right panel]. A real-time digital frequency domain FLIM system using FPGAs at 250  MS/s was implemented by Serafino et al.^7^ that is capable of simultaneous three-channel detection using avalanche photodiodes (APD).

### 2.2 Wide-Field FLIM

Wide-field FLIM can image a larger field of view (FOV) by employing single-photon avalanche diode (SPAD) arrays or SPAD detectors with complementary-metal-oxide semiconductor (CMOS) technology, which has gained popularity in the last year. SPAD arrays can have temporal resolution in picoseconds and single-photon sensitivity that is advantageous for lifetime measurements. Figure 1(b) shows a schematic of a SPAD array. Lagarto et al. used a SPAD array (32×64 SPADs) detection scheme coupled with a fiber-optic probe for real-time FLIM and spectral imaging on a macroscopic scale.^8^ Lifetime was estimated using the phasor approach in real time using time-correlated single-photon counting (TCSPC). Additionally, multispectral FLIM was implemented by Ghezzi et al.^9^ using an 18×1 linear SPAD array. Moreover, Zickus et al.^10^ aimed for real-time FLIM using a time-gated SPAD array capable of achieving widefield FLIM of 500×1024  pixels with lifetime estimation performed by a custom artificial neural network (ANN) that was trained on simulated lifetime decay curves. Given the advantages of the SPAD array (Table 1), this technology is likely to increase in popularity for FLIM.

**Table 1:** Advantages and limitations of recent advances in FLIM instrumentation and analysis.

Technique	Advantage	Limitation
**FLIM instrumentation**		
Fast FLIM techniques	Visualizes fast dynamics	Requires additional electronics
Wide-field FLIM with SPAD arrays	Faster image acquisition from large samples	Microlens arrays needed to mitigate the fill factor of SPAD arrays
		Time-gating mode requires special noise-optimized processing and time-to-digital converters with onboard TCSPC electronics^17^
Single molecule FLIM	Super-resolution FLIM images	Requires additional data analysis (e.g., pattern matching)
Light-sheet FLIM	Volumetric visualization of large samples	FLIM cameras are in early stages of development so photon detection efficiency is still low
Fiber-optic clinical FLIM	Enables clinical applications	Fiber transmission loss
**FLIM analysis**		
Cell classifiers	Provides biological relevance	Requires expert annotation
Pixel-level analysis	Multidimensional data analysis at high resolution	Some methods require significant training and computational time
Community resources	Accessible to non-experts	Difficulty comparing data across platforms

### 2.3 Single-Molecule FLIM

A super-resolution microscope that can also acquire lifetime data is advantageous for detecting fluorophores with similar spectral properties and for lifetime-based Förster resonance energy transfer (FRET) measurements. Thiele et al.^11^ combined a single-molecule localization microscopy (SMLM) technique such as direct stochastic optical reconstruction microscopy (dSTORM) and DNA points accumulation for imaging in nanoscale topography (DNA-PAINT) with FLIM [Fig. 1(b)]. Data were collected using a single APD and TSCPC electronics to generate two-channel images. Super-resolved separation of two species was achieved by Baysian pattern matching on confocal FLIM data. Figure 1(c) shows schematics of single-molecule FLIM with representative lifetime decays from two single-molecule localizations. This is the first time that super-resolved single-molecule localization FLIM was demonstrated, which provides a simpler alternative to point-spread function engineering methods such as stimulated emission depletion (STED). In a subsequent work, this group implemented a TCSPC-camera-based wide-field single-molecule localization approach for super-resolution imaging using a similar pattern matching method.^12^

### 2.4 Light-Sheet FLIM

FLIM with a light-sheet illumination scheme enables volumetric lifetime data acquisition in large samples [Fig. 1(d)]. Hirvonen et al. merged wide-field TCSPC FLIM with light-sheet microscopy and microchannel plate detectors with a delay-line position sensitive anode.^13^ More recently, a digitally scanned laser light-sheet microscope with FLIM capabilities was implemented by Li et al.^14^ In this work, a gated optical intensifier (GOI) with a sCMOS camera was used for wide-field time-gated imaging.

### 2.5 Fiber-Optic Clinical FLIM

Fiber-optics-based lifetime imaging enables FLIM for clinical applications. Marsden et al. presented a novel lifetime system based on fiber-optics excitation and detection. The point measurement system can be used intraoperatively and is capable of real-time processing (>30 frames per second).^15^ A similar real-time fiber-based system, simplified schematic shown in Fig. 1(e), was also implemented by Lagarto et al.^16^ that combined TSCPC detection and phasor analysis.

Table 1 summarizes some of the advantages and limitations of the instrumentation discussed.

## 3 New Approaches to Analyze FLIM Data

### 3.1 Cell Classifiers

**Fig. 2:**
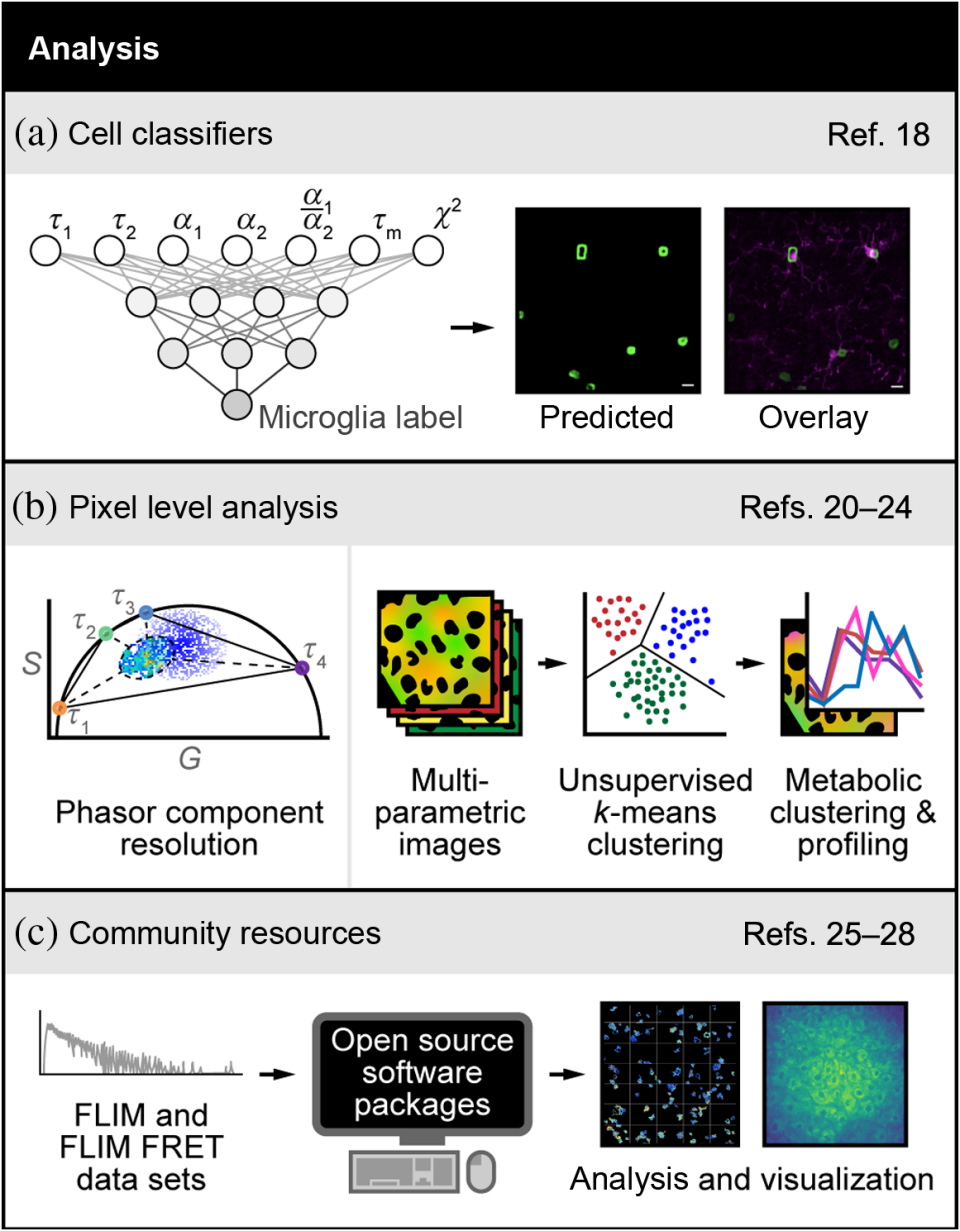
Advances in FLIM analysis. (a) FLIM of the intrinsically fluorescent metabolic co-factor nicotinamide adenine dinucleotide (phosphate) [NAD(P)H] was combined with ANN-based machine learning to differentiate microglia from other glia cell types in the brain. The two-exponential decay lifetimes $\left(\tau_{1},\tau_{2}\right)$, weights $\left(\alpha_{1},\alpha_{2}\right)$, mean lifetime ($\tau_{m}=\alpha_{1}\tau_{1}+\alpha_{2}\tau_{2}$), and goodness-of-fit ($\chi^{2}$) were used as input parameters to identify microglia in cell culture mixed with other glial cells and in fixed brain tissue. Adapted with permission from Ref. 18. (b). Left panel: phasor plots of fluorescence decays provide a visual distribution of fluorescence lifetimes and are derived from a Fourier transformation of the fluorescence lifetime decay with unitless horizontal (G) and vertical (S) axes.^19^ Here the phasor method was used to resolve the decay in each pixel of an image in live cells or mouse liver tissues with two or more exponential components without prior knowledge of the values of the components. Adapted with permission from Ref. 20. Right panel: spectral and lifetime imaging of NAD(P)H and FAD was combined with unsupervised k-means clustering of each pixel to identify regions within cells that have almost uniform metabolic properties. This method detects the cellular mitochondrial turnover and the metabolic activation state of intracellular compartments at the pixel level. Adapted with permission from Ref. 22. (c) FLIM and FRET decays acquired through any method can be analyzed with open-source software packages to quantify molecular changes and render images. These open-source packages include community forums to educate non-experts and foster continuous development by experts. Images adapted with permission from Refs. 26 and 27. References to all relevant papers for each section have been noted.

Several recent papers have developed machine learning tools to analyze FLIM images. Some methods use standard labels to serve as ground truth to train algorithms that recognize specific cell types based on FLIM. For example, Sagar et al.^18^ used an ANN to differentiate microglia from other glia cell types in the brain based on label-free FLIM of reduced nicotinamide adenine dinucleotide (phosphate), NAD(P)H [Fig. 2(a)]. Their approach identified microglia in cell culture mixed with other glial cells with a true positive rate >0.9. They also identified microglia in fixed brain tissue with a true positive rate of 0.79. This is an important contribution because microglia are challenging to identify as most fluorescent labeling approaches cross-react with other immune cell types, are often insensitive to activation state, and require the use of multiple specialized antibody labels.

### 3.2 Pixel-Level Analysis

Alternatively, pixel-level analysis methods can be used to classify molecular features in a FLIM image. The phasor approach plots the distribution of fluorescence lifetimes in a two-dimensional frequency space, which provides an intuitive method to group pixels that belong to molecular species of similar lifetimes.^19^ This approach has recently been expanded to automatically group pixels for improved accuracy and unbiased analysis [Fig. 2(b), left panel].^20^ Specifically, the decay in each pixel of an image with two or more exponential components was recovered without prior knowledge of the values of the components using a graphical method for two components and a minimization method for three components. This specific use of the phasor approach is attractive because it can disentangle species with different lifetimes and spectra within the same pixel.

Like the phasor approach, machine learning has been used to achieve fit-free estimates of fluorescence lifetimes from decays on the pixel level,^21^ where simulated data were used to compare a machine learning approach to standard fitting algorithms. The root-mean-squared-error, which represents the difference between the reconstructed and measured decay traces, was observed to be lower for the machine learning approach compared to the standard fitting algorithm, including up to three-component analysis.

Fit-free analysis methods have also been developed for spectral-lifetime images. K-means clustering was used to cluster subcellular pixels with metabolically distinct properties using spectral and lifetime imaging of autofluorescence [Fig. 2(b), right panel].^22^ Two distinct clusters were identified as regions that have almost uniform metabolic properties, including the cytosolic activation state and the mitochondrial activation state. Inhibition of glycolysis and the electron transport chain confirmed the accurate isolation of these metabolically distinct clusters. Furthermore, deep learning methods have been developed to disentangle spectral and lifetime information on a pixel-wise basis. Intes et al.^23^ unmixed spectral and lifetime data using the unmix multiple emissions (UNMIX-ME) algorithm. The algorithm was trained and validated *in silico* and benchmarked against a conventional least-squared lifetime fitting method for three- and four-exponential decays. UNMIX-ME was then used to monitor near-infrared FRET interactions *in vitro* and *in vivo*. The same group developed a convolutional neural network for spectrally resolved fluorescence lifetime imaging with compressed sensing.^24^ This high compression enables *in vivo* spectral-lifetime imaging at enhanced resolution, acquisition, and processing speeds, without the need for experimental training datasets. This approach was validated *in silico* and demonstrated *in vivo* and *in vitro* with acquisition times reduced from 2.5 h to 3 min at 99% compression.

### 3.3 Community Resources

One important component of FLIM analysis is dissemination to the community, standardization across labs so that results can be compared, and integration of FLIM analysis with image and data processing [Fig. 2(c)]. To address these needs, Gao et al.^25^ created FLIMJ, an ImageJ plugin and toolkit that provides accessible image analysis workflows with FLIM data. FLIMJ offers FLIM fitting routines with seamless integration with many other ImageJ components and the option to extend features to create complex FLIM analysis workflows. FLIMJ routines can also be used with Jupyter notebooks and integrate naturally with science-friendly programming (e.g., Python and Groovy). FLIMJ was demonstrated on lifetime-based image segmentation and image colocalization with comparisons to standard FLIM analysis tools. Similarly, a Python software package named flimview was developed to read, explore, manage, and visualize FLIM images.^26^ The flimview package is open source, with test data and documentation available on Github. Other open source FLIM analysis software includes FLIMfit, which is an OMERO client to analyze and visualize FLIM data,^27^ and FLIM-FRET analyzer, which provides automated analysis of lifetime-based FRET images.^28^

Table 1 summarizes some of the advantages and limitations of the analysis techniques discussed.

## 4 Recent Applications of FLIM in Biology and Medicine

Numerous studies have used FLIM to understand molecular features of biological systems and changes due to disease progression or drug treatment. Below are a few examples of autofluorescence FLIM, FLIM of exogenous molecular probes, and FLIM-FRET in the past year.

### 4.1 Autofluorescence FLIM Applications

**Fig. 3:**
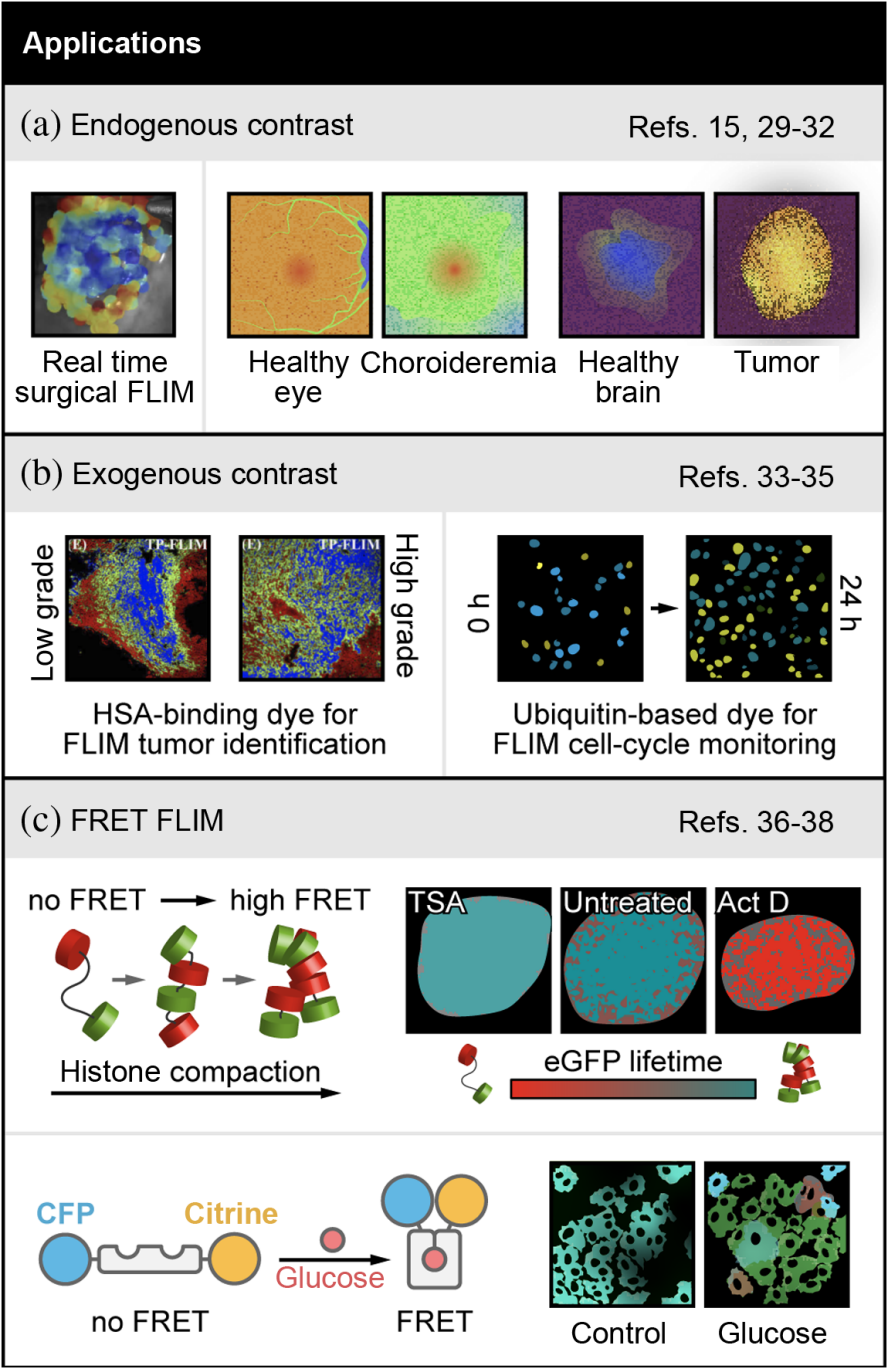
Examples of FLIM in biology and medicine. (a) Left panel: endogenous contrast applications include automated FLIM visualization using FLIMBrush on a clinical head and neck surgery case. Adapted with permission from Ref. 15. Middle and right panels: FLIM detection of choroideremia (middle)^30^ and brain tumors (right)^31^ are shown with stylized images that represent changes observed in original publications. (b) Left panel: applications with exogenous contrast include quantitative analysis of HSA concentrations in cryosection from a low-grade and high-grade serous ovarian cancer patient, using the concentration dependent squarine dye (blue = high [HSA], green = mid [HSA], red = low [HSA]). Adapted with permission from Ref. 34. Right panel: FLIM of an ubiquitination-based dye, FUCCI-Red, which is a lifetime sensor of cell cycle state (blue=S/G2/M, green=G1/S, yellow=G1) shown with stylized images representing changes observed in original publication^35^ (c) Top panel: FRET-FLIM applications include a new FLIM-FRET pair sensitive to chromatin compaction. Adapted with permission from Ref. 36. Pseudocolored chromatin compaction maps of the cells in the top row according to the palette defined below scaled between 2.5 ns (teal, 0% FRET, donor lifetime) and 2 ns (red, 21% FRET, quenched donor). Bottom panel: intracellular glucose concentration imaged with a FLIM-FRET pair.^38^ A shift toward red indicates higher intracellular glucose, which can be seen in the glucose condition represented using stylized images. References to all relevant papers for each section have been noted.

FLIM of autofluorescence is attractive for clinical use because it provides label-free images of tissue function. For cancer surgical guidance, Marsden et al. have developed a promising integrated fiber-based autofluorescence FLIM and analysis tool, which can spectrally resolve collagen, nicotinamide adenine dinucleotide (NADH), flavin adenine dinucleotide (FAD), and porphyrins. This tool called FLImBrush can be used in the operating room to detect cancer margins in a label-free manner and uses a deep learning-based image segmentation, block-matching-based motion correction, and interpolation-based visualization. This allows for real-time imaging and image processing to enable intraoperative imaging and surgical guidance, as demonstrated on surgeries of head and neck cancer patients [Fig. 3(a), left panel].^15^ Additional work to improve the overlay of the real-time FLIM images on the surgical field will yield an exciting new tool for image guided surgery. Another application, fluorescence lifetime imaging ophthalmoscopy (FLIO) has been in clinical use for 20 years.^29^ Recent advances by Vitale et al. show that FLIO can detect an increase in autofluorescence lifetime in the diseased tissue of a small cohort of patients with choroideremia, an ocular disease, compared to normal surrounding tissue^30^ [Fig. 3(a), middle panel].

Autofluorescence FLIM of *ex vivo* tissues have also been investigated this year. Erkkila et al. showed that FLIM of NAD(P)H, and protoporphyrin identify tumor margins in low- and high-grade human gliomas^31^ [Fig. 3(a), right panel]. With a larger sample size, this promising result could yield another useful imaging-based method for intraoperative guidance. Additional work by Bower et al. imaged changes in NAD(P)H lifetimes and calcium transients in *ex vivo* mouse brains and showed that there are significant differences in the NAD(P)H lifetime dynamics between neurons and astrocytes.^32^

### 4.2 FLIM of Exogenous Molecular Probes

New studies have generated molecular probes with fluorescence lifetimes that are sensitive to physical conditions, including new FLIM probes for protein concentration and cell cycle. Yi et al. developed a small squarine dye with a fluorescence lifetime that changes depending on serum albumin concentrations.^33^ Recent work in human ovarian cancer biopsies by Lin et al.^34^ shows that this dye is sensitive to concentrations of human serum albumin (HSA), which is a protein that is often concentrated near solid tumors [Fig. 3(b), left panel]. More importantly, the dye shows differences in the HSA microenvironment between low- and high-grade serous ovarian cancer in humans.

A novel fluorescence lifetime probe, FUCCI-Red, relies on the different fluorescence lifetimes of the red proteins mCherry and mKate2.^35^ This all-red probe is an advancement over the standard FUCCI probe that uses fluorescence spectral shifts between multicolored fluorescent proteins to detect cell-cycle phases, thereby limiting the color channels available for multiplexed imaging. The new FUCCI-Red capitalizes on lifetime shifts and enables multiplexed detection of cell cycle information alongside autofluorescence FLIM on the single-cell level [Fig. 3(b), right panel].^35^

### 4.3 FLIM-FRET Applications

Finally, FLIM-FRET can capture extracellular and subcellular interactions on the nanoscale, including several studies this year. For example, an FLIM-FRET pair of eGFP and mCherry, both tagged to histones, detected changes in chromatin architecture after induction of DNA damage.^36^ This FLIM-FRET method provides spatiotemporal visualization of chromatin compaction-dynamics in living cells, which is technically difficult without the use of super resolution microscopy because of the very small spatial scale on which chromatin compaction occurs [Fig. 3(c), top panel].^36^^,^^37^ The FLIM-FRET method makes this analysis more accessible to labs that do not have super resolution microscopes. Another novel FLIM-FRET detector uses an eCFP-Citrine FRET pair tagged to a bacterial protein that changes conformation upon binding to glucose [Fig. 3(c), bottom panel].^38^ This complex can be stably expressed in breast cancer cells to detect single-cell heterogeneity of intracellular glucose concentrations, under multiple culture conditions *in vitro* and *in vivo*. Intracellular glucose imaging of single cells provides an exciting new tool to investigate metabolic heterogeneity and its effects on treatment response.

## 5 Conclusions

FLIM is a widely used tool for biomedical imaging and has advanced the field of microscopy in the past few decades. In this perspective, we provided updates of innovations in FLIM within the last year that we as experts in the field found compelling. These new developments have been categorized into advances in instrumentation, new approaches to analyze FLIM data, and recent applications of FLIM in biology and medicine. Recent updates to FLIM instrumentation include faster acquisition speed, improved spatial resolution, and translation into the clinic. Rapid advances in algorithms for FLIM analysis in the past year aim to improve image segmentation, analysis speed, quantify multidimensional heterogeneity, and perform multiparametric investigation. Additionally, there is an emphasis on open source FLIM analysis tools that can be integrated into other data processing pipelines. These computational tools unravel spatial and molecular features of cellular physiology that are not apparent with qualitative observation of FLIM images. Some of the advantages and limitations of these new instrumentation and analysis techniques are summarized in Table 1. Finally, autofluorescence FLIM has impacted clinical care, while FLIM coupled with fluorescence lifetime probes can quantify chemical and physical changes to molecules including changes in concentration, cell cycle, glucose, and others. Overall, the numerous high-quality publications this year indicate that FLIM technologies, analysis, and applications will continue to develop, gain popularity, and impact biomedical research.
